# Evidence-based decision-making in a climate of political expediency: insights from local government

**DOI:** 10.1177/17579139241256879

**Published:** 2024-06-10

**Authors:** J Woodall, C Homer, C Freeman, J South, J Cooke, J Holliday, A Hartley, S Mullen, B Stafford

**Affiliations:** Leeds Beckett University, City Campus, Portland Way, Leeds LS1 3HE, UK; Sheffield Hallam University, Sheffield, UK; Leeds Beckett University, Leeds, UK; Leeds Beckett University, Leeds, UK; The University of Sheffield, Sheffield, UK; Mid Yorkshire Hospitals NHS Trust, Wakefield, UK; Wakefield Metropolitan District Council, Wakefield, UK; East Riding of Yorkshire Council, Beverley, UK; The University of Sheffield, Sheffield, UK

**Keywords:** policy, evidence, local government

## Abstract

**Aims:**

Local authorities in England are responsible for public health and health promotion. This article sought to explore how research and decision-making co-exist in a local authority in England.

**Methods:**

An Embedded Researcher was based within the local authority and used qualitative methodology to address the research aim. Interviews and focus groups were employed to ascertain a range of stakeholder views in the local authority. All transcripts were coded on NVivo 12 by the Embedded Researcher and two members of the research team cross-checked a sample for coding accuracy. Data were analysed using framework analysis.

**Results:**

The data suggest several barriers to using research to inform decision-making in health promotion and public health. The study shows that research is valued in local authorities, but not always privileged – this is due to cultural factors and practical political reasons which often means that decisions need to be made expediently. Participants outlined a juxtaposition between academic credibility; timeliness to complete the research and the financial cost associated with it; against the independence and credibility that independent academics could bring.

**Conclusion:**

Policy formulation and delivery is an integral aspect of health promotion and critical to achieving improved population health and reductions in health inequalities. However, there exists tensions between gathering research evidence and making research-informed decisions. The article concludes by advocating the use of Embedded Researchers to fully understand how research is gathered and used to support public health and health promotion policymaking.

## Background

Effective policymaking is one of the key resources in improving public health, reducing health inequalities and fostering supportive environments.^1,2^ However, in a previous publication, authors discussed a tension between gathering research evidence and making research-informed decisions in local government in England in relation to population health and health promotion. We focus on the tension between rigour in research and making political decisions in an expedient and timely way. A conflict was identified between political systems – which frequently require decisions to made swiftly – and academic rigour and process, which can be time-consuming. If we are to promote research-use-in-context, then we need to harness and use such tension and work with it. Drawing on data from a wider research study in one local government area in the United Kingdom, which optimised the use of an Embedded Researcher (ER) to explore and understand this tension more clearly, this article aims to explore how research and decision-making co-exist in a local government area. In doing so, it offers potential solutions to aid both researchers and policymakers working in this context.

The research here focuses on England where local government is a key constituent for addressing health inequalities and influencing the health of individuals and communities.^3^ Local government has been regarded as an effective vehicle to tackle the multiple determinants of health, as opposed to health systems with their focus on a medical model, but there are concerns that generating and using research evidence to inform decision-making and action is a challenge.^4–6^

There is appetite to strengthen evidence use in local government^7^ and an accompanying body of research that has explored decision-making in local government vis-à-vis public health and health promotion. There are themes around the reliance on anecdote to support decision-making and how this can be problematic when it is the only source driving decision-making and the value of economic analysis when justifying public spending.^8^ Practically, there are also considerable challenges of using evidence effectively in local government, including: organisational churn, rising demands, siloed thinking and the time and space for staff engagement.^7^ The relationship of health promotion with ‘evidence’, however, has been explored in detail. The relationship has been described as an uncomfortable one because of the constant comparison with the principles of evidence-based medicine.^9^ Some have even suggested that the inability to have a coherent understanding of what evidence is and means for health promotion has been a significant barrier for practitioners in undertaking evaluation of their practice.^10^ Despite this, evidence-based health promotion has been defined^11^ (p. 141) as: ‘the systematic integration of research evidence into the planning and implementation of health promotion activities’. Indeed, one of the key ‘activities’ in public health and health promotion is the effective policy formulation.^2^

The notion of ‘evidence’ in policy and decision-making has been debated for some time. Evidence can comprise a wide range of sources – some routinely gathered by local government, others produced by academics or by organisations, such as NICE (National Institute for Health and Care Excellence) and OHID (Office for Health Improvement and Disparities) or the LGA (local government association). Recently, the notion of ‘evidence-informed decision-making’ (EIDM) involving the best available research evidence with contextual factors, including community preferences, local issues (e.g. health, social), political preferences and public health resources has become more accepted.^12^ Nevertheless, policy decisions can frequently be underpinned by political timeliness (based on perceived short-term opportunities and political preferences) or mandates from central government, rather than credible research evidence.^1^ It may be that these decisions are necessary, due to a lack of available evidence. However, we argue here that these relationships need greater exploration as it suggests that decisions on public health resourcing may be being made on evidence that is politically timely and preferred rather than academically sound. This could be for a whole myriad of reasons which the article seeks to explore further.

## Methodology

The research focused on a single local authority in the north of England. In England, local authorities are responsible for public health and health promotion in their communities. The positioning of public health in local authorities has been broadly welcomed given the shape services to meet local needs, influence the wider determinants of health and to tackle health inequalities.^13^ The local authority where this research was focused is in the top 20% of the most deprived districts in England and on average, people die younger than in other parts of England. Cardiovascular, cancer and respiratory illnesses are in high levels in the district resulting in people becoming ill at a younger age and having to live with their illnesses longer compared to most of the rest of the country.^6^

Data collection was undertaken by an ER who was based within the local authority during the three-month study period. The ER model places a researcher in a non-academic organisation to better link research and practice.^14,15^ The rationale to use an ER in this study was to gain depth and richness in data through having a researcher integrated within the culture and environment. However, this was compromised during the COVID-19 pandemic and the ER became digitally, rather than physically, embedded in the local authority. As part of the ER process, a co-applicant of the study facilitated access for the ER to attend online team meetings at the operational and strategic level with various departments across the local authority to meet employees, develop a rapport with teams and raise awareness of the study. This included attending team meetings and formal committees. The ER was introduced to strategic directors by another co-applicant of the study who was also a member of the local authority leadership team.

Interviews and focus groups were the primary research method for ascertaining data. Purposive sampling was used to identify key individuals in the local authority. The sampling was conducted with support of the project steering group in which a discussion had to identify the key strategic roles and groups from across the authority that would need to be included. The steering group also identified groups of people who were research-active (involved in delivering or commissioning research or who held a research-related qualification), roles within the public health department where research was considered to be used in practice on a regular basis and elected members (i.e. local politicians) who had responsibilities for different portfolios across the local authority. Participants were recruited via email invitation. Consultation with the study steering group informed the sampling of three focus groups which were conducted with: Focus Group 1 – Elected Members (*n* = 3), Focus Group 2 – Public Health Officers (*n* = 6) and Focus Group 3 – Officers with research interests across the local authority (*n* = 4). Interviews (*n* = 7) were conducted by the ER with Corporate Directors and Service Managers purposively sampled to enable the research questions to be explored fully. All aspects of the study received ethical approval from Sheffield Hallam University and Leeds Beckett University and access permissions were gathered from the local authority via the strategic leadership team.

Interviews and focus groups were conducted simultaneously during the study. These lasted between 30 and 60 minutes. Interviews and focus groups explored a range of issues which were informed through the Research Capacity Development (RCD) framework developed by Cooke.^16,17^ Using the principles of the RCD framework, the interview schedules and focus group guides covered the following: linkages and partnership; skills and confidence in the workforce and wider community; infrastructure of the council and wider partnerships, research use and dissemination, experience and assets of co-production in projects (including citizen and public engagement in projects); and ownership, leadership and sustainability of research type of activity.

## Data Analysis

Interview and focus group recordings were transcribed by an external transcription company, anonymised and shared as a secure online file which was accessible by three members of the research team. All transcripts were coded on NVivo 12 by the ER and two members of the research team cross-checked a sample for coding accuracy. Data were analysed using framework analysis.^18^ Framework analysis was used as an expedite method given the short timescale for the project funding and was deductively informed using the work of Cooke.^16,17,19^ Specific elements of the RCD framework were used in the development of the matrices – a core aspect of framework approach – this seemed pragmatic in deductively analysing the data set given the RCD framework was used to inform the data collection tools, as discussed earlier. Given the limited timeframe set by the funder for the research delivery, the data were analysed sequentially with interview analysis being completed first, followed by focus groups. This was based on pragmatics but was beneficial in refining analytical categories and themes during the process and supported the triangulation of the two sets of data. Inductive coding and inductive thematic development were also part of the analytical process to enable specific ‘local’ issues within the local authority to be represented.

## Findings

This section presents four thematic areas derived from data analysis, using illustrative quotations where appropriate to exemplify key points.

### Research informing the work of the local elected representatives

All participants were acutely aware of the democratic nature of the organisation and were well-versed in the decision-making process and how their respective roles contributed to this. Connection with the constituents was critical, using research approaches to ascertain their perspectives:

*I think it’s seen as a democratic organisation, that we’re closer to the people in that respect. We’ve got democratically elected local representatives which are really tuned into that local voice and want to make sure we’re representing that local voice. So I think it’s very important to local authorities to be seen to get that. It’s a key factor in key decisions.* (Focus Group 3 – Officers with research interests across the local authority)

Elected politicians in the authority who participated in the study fully supported the notion of listening to the community and mentioned some ways of accessing this through surveys and conversations. The importance of research in gathering those views or indeed adding further depth and insight, however, was questioned:

*As elected members we compile surveys within our communities and as part of specific roles we hold as councillors. We speak with staff and residents alike and ensure the community voice is heard within our roles. We are democratically elected by the public and for me, it is the most important thing to ensure their voices can be heard. I do, however, think that the awareness of the importance of research needs raising across the organisation.* (Focus Group 1 – Elected Members)

It was understandable that elected politicians representing a constituency wanted local information to meet and address local needs and challenges. Some participants working in the local authority challenged some aspects of research that may not take into account local context and instead have a more distil focus on issues that may not be central to local debate:

*That’s what members are interested in really, what the local perspective is, not so much about what the national or international data or analysis will be, so as long as it can be made relevant and local I think it lands well.* (Focus Group 3 – Officers with research interests across the local authority)

### Political pressures inhibiting quality evidence production

One salient theme in the data was the political pressures which could work in opposition to quality research design and data analysis. While the political climate could be facilitative of research, there was a sense that it could often work against it:

*. . .sometimes politics and research meet in a way that’s positive and constructive, and sometimes it collides.* (Corporate Director)

The demand to produce evidence ‘quickly’ was a common thread with public health practitioners. Staff were cognisant that the research was frequently being produced for local political figures who were conscious of delivering their mandate within their electoral cycle. It seemed that research planning needed to be acutely interlinked with the policy timetables of local elections:

*It’s understanding the timescales and it’s sometimes you may be asked to look at a problem and they’re expecting a solution very, very quickly; whereas for quality research it’s going to take a prolonged period of time. Obviously within local authority we tend to work in four-year cycles really, if that, coming towards elections and things like that. So it’s understanding that things don’t happen overnight and that if you want quality information, quality data, it’s going to take time to collect before the solutions can even be dreamt up.* (Focus Group 2 – Public Health Officers)

The political pressure not only meant that research had to be undertaken quickly but also meant that the focus of the research or the policy design could be wrong. Instead of focusing on the root causes of issues within the local authority – which could be complex and multifactorial – there was encouragement to look at the issues manifesting from those problems:

*There’s a political element to this because there’ll be a pressure from members to be seen to be moving quickly. . .I think that all results in us wanting to always deal with the immediate symptom of whatever issue we’re trying to address and doesn’t give us the time to really understand the cause or factors because to do that that takes time and work and often research. And then, therefore, we end up addressing the wrong thing.* (Focus Group 1 – Elected Members)

Research and subsequent policy action in relation to poverty was demonstrative of the tension between ‘quick wins’ for local elected officials and meaningful change to tackle systemic issues impacting on poverty in the area:

*How do you balance short-term alleviation versus long-term systemic shifts? And political drive will always be for more of the short-term because election, you know, from election to election it’s about immediate response; whereas actually the more value is actually moving people out of poverty rather than making them feel more comfortable whilst they’re in it. But some of that research and some of that evidence and things that support that longer-term view might be at odds with some of that short-term view.* (Focus Group 2 – Public Health Officers)

Often practitioners were put into a situation where they were having to trade-off between finance and current political priorities and undertaking rigorous research:

*I’m doing a piece of research and I’ve got to balance this between the need to do it, make it robust research that actually makes a valuable point, but also the need to get it done before the end of the financial year so that we can spend the money this year, otherwise that budget will have gone and the research has been a complete waste of time because priorities will have changed.* (Focus Group 2 – Public Health Officers)

However, many of those using and generating research in local authorities were comfortable with the parameters of research in a political context and did not find it problematic:

*Politics doesn’t always treat proper research with the objectivity that it warrants. And that’s, I would say from my point of view, that’s neither fortunate or unfortunate, it’s just a reality of the operating context for politically-led organisations.* (Corporate Director)

### Research: ‘a little bit academic and a little bit of non-delivery’

There was a cultural perception by some participants that research was not linked to ‘action’ and ‘delivery’ essential to a functioning local authority. Some participants noted that the local authority was characterised as being pragmatic and operational which was perceived to be at odds with research:

*I think councils tend to be quite pragmatic and operational and probably less. . .around the academic research side.* (Focus Group 3 – Officers with research interests across the local authority)

*Historically I think there’s a view that research is not doing. So we’ve become a council that is overly focused on action rather than consideration and careful development of those actions.* (Focus Group 1 – Elected Members)

There were some exceptions to this viewpoint – participants working in public health roles in the local authority described research as being fundamental to their practice and moreover embedded in their work. That said, some participants conceptualised research in their areas of work as being a ‘luxury’ rather than a core part of professional practice.

### Juxtaposing rigour and the timeliness of evidence from academic institutions

Making policy decisions that support communities was critical to the role of the elected politicians and, moreover, central for the local authority’s remit in supporting this decision-making process. Research commissioned and delivered by academic institutions was often seen as being a barrier to timely decisions being made. This was notably due to the time needed for academic research to produce robust conclusions:

*. . . the timescales to actually do something that is academically rigorous, that takes so long to do that it’s a complete and total waste of time, because by the time you get the results the political landscape has moved on.* (Focus Group 2 – Public Health Officers)

The tensions between timeliness, cost and academic rigour and credibility were frequently discussed and explored during data gathering. The majority of participants suggested that rigour, impartiality and credibility were key elements of delivering research of sufficient quality to informal local decision-making. There was some suggestion that research was worth waiting longer for, if it was to be a high-standard:

*I think you asked the question around what the value is for the research with universities, and, for me, I think it gives credibility, I think that’s probably the key one, from an academic institute, and impartiality I suppose as well. It gives that sense of both those factors I think.* (Focus Group 3 – Officers with research interests across the local authority)

In contrast, in-house research delivery was challenged for its potential to be ‘limited’ in data analysis or presentation, perhaps due to a lack of training, skills or capacity. There were some concerns that in-house research production had the potential to be influenced by outside factors:

*You know, it’s not a council officer doing this research to present a paper up to elected members, you know, it’s somebody who’s come in, they’re independent and this is what that research is telling them, there should be no other influences there.* (Focus Group 2 – Public Health Officers)

## Discussion

This article sought to further understand the application and use of research evidence in decision-making processes in one local authority. The article draws on a range of perspectives from various stakeholders in the local authority, drawing on an ER model, which we reflect on later, to gain rich and detailed insight.

The relationship between evidence, and its myriad of forms, and decision-making processes in local government was demonstrated through the data gathered. Elected politicians were cognisant of the importance of research to inform their choices and strategies and relied on accessing the community perspective to do so through surveys or through more informal data gathering. Some studies have suggested how elected politicians working in local authorities rely on rudimentary internet searches to find information to inform their practice – often due to the inaccessibility of peer-reviewed research publications.^20^ In addition, barriers to accessing research that is contextually specific to the local area are a common problem that elected politicians face when using evidence to inform decision-making.^21^ Accessing local community viewpoints, therefore, seemed a sensible strategy for elected politicians but how this interconnected with empirical research was unclear and not discussed.

The study has demonstrated that the political context in which people were working created challenges on creating and interpreting research evidence. It is common to see short-term decisions made in health promotion practice that might provide ‘short-term’ gains rather than sustained longer-term change in the period of a political cycle.^2^ Lifestyle drift can, therefore, occur where practice focuses on the manifestation of deeper health and social inequalities.^22^ Similar patterns were observed in this study where research was often used to facilitate immediate solutions to local problems rather than tackling systemic problems which could be more time-consuming and complex. This finding has been noted in other studies of local government^23^ but it may also be the case that top-down instruction from central government or budget restraints might also be a contributing factor to short-term policymaking.

There was a broader view that research was a ‘luxury’ in local government and not regarded as embedded in daily practice. A research culture and the organisational culture of the local authority was regarded as being, at times, at odds with one another. The daily business of local authorities was on delivery for local people and being pragmatic, yet research was somehow perceived to be an inhibitor to this. Perhaps this conceptualisation of research as being an inhibitor and luxury stems from a wider misunderstanding about the plethora of research strategies and methodology – many of which could embody action research principles that could directly support decision-making in public health rather than detract from it.^24^ Similarly, the view that research took too much time to undertake and would not be expedient enough to facilitate decision-making, seemed a notion that many subscribed to.

Participants outlined a juxtaposition between academic credibility; timeliness to complete the research; and the financial cost associated with it; against the independence and credibility that independent academics could bring. Overall, there was a sense that in an ‘ideal scenario’, independent research would be undertaken as ‘in-house’ research could be open to influence by wider political factors. The relationship and merits of ‘in-house’ *versus* externally commissioned research to inform local authority decision-making in public health has not been fully explored in the literature. There seems, however, to be a number of options open to local authorities. First, ‘in-house’ research which can enable studies to be undertaken within geographical context and therefore to be highly valued by local decision-makers. The obvious drawback here is the scale of the resources involved. The staff of such an ‘in-house’ unit would need to be quite highly skilled in a range of research methods and offered competitive employment contracts which include career progression. The severity of the present and prospective financial constraints on local authorities is likely to make these costs prohibitive.^25^ Second, using existing evidence already produced in peer-review publications. This brings challenges in relation to access and interpreting the data meaningfully for the specific local context.^6,24^ However, it can mean that the information has already been produced which means that evidence-based decisions can be made in a very timely manner with little cost implication (especially if the research is published in an open access journal). Third, commissioned research by academic institutions – the major benefit of this source is likely to be the provision of evidence which is most closely focused on the issue facing the authority and which takes the account of the distinctive socio/economic/cultural characteristics of the local authority area. That said, the severe financial constraints on local authorities already mentioned may make this option less likely.^25^

Finally, while further evaluation of the role is required, the richness of findings generated through this study would point to the usefulness of embedded research to the generation of academic research in a timely, cost-effective and rigorous way. Notwithstanding the potential drawbacks that such a model can entail, this approach has growing momentum in the field of health promotion research given the close relationship necessary between research and practice.^15^ ERs usually straddle two organisations – one practice organisation and one academic organisation^14^ and have the ability to positively influence both. The embedded nature of the researcher means that ultimately, they are ‘responsive and agile’ both to seizing opportunities for data gathering but also in producing outputs that are practice-oriented and linked intimately with what is required at a local level.^6^

## Conclusion

This article sought to explore how research and decision-making co-exist in a local authority. It has highlighted the complexity of decision-making and show how research influences the process. The study shows that research is valued but not always privileged in local authorities – this is due to cultural factors and the political expediency which often means that decisions need to be made quickly. The article argues that research-informed decision-making is helpful in itself but also that the development of research skills and understanding by policymakers is also helpful for their role. Of course, findings from this study should be tested further as local authorities are highly heterogenous and indeed the focus here was specifically on health and not wider issues in the local authority where stronger research traditions may, or may not, exist.^20^ Moreover, due to COVID-19, the ER in this study was digitally, rather than physically, embedded and this may have limited some aspects of the integrated nature of the approach. That said, it is clear how ERs can help drive cultural change in both institutions and foster collaborations with other key agencies and think-tanks in developing approaches to research that are in sympathy with local government. This study was based in one local authority in England, but the finding supports international studies which have drawn similar conclusions on policymaking in public health both in local and national government;^1^ further research would be of value to tease out further specific difference or similarities in the use of research evidence in decision-making at all governmental levels. The article has suggested a number of ways in which research can play a more integral part of local authority decision-making, including the exploration of using ERs.

## References

[1] Van de Goor I Hämäläinen RM Syed A , et al. Determinants of evidence use in public health policy making: results from a study across six EU countries. Health Policy 2017;121(3):273–81. 10.1016/j.healthpol.2017.01.003 PMC5754321 28139253

[2] Woodall J Cross R . Essentials of health promotion. London: Sage; 2021.

[3] Local Government Association (LGA). A matter of justice: local government’s role in tackling health inequalities. London: LGA; 2018.

[4] Lorenc T Tyner EF Petticrew M , et al. Cultures of evidence across policy sectors: systematic review of qualitative evidence. Eur J Public Health 2014;24(6):1041–7. 10.1093/eurpub/cku038 24681818

[5] Oliver K Innvar S Lorenc T , et al. A systematic review of barriers to and facilitators of the use of evidence by policymakers. BMC Health Serv Res 2014;14:2. 24383766 10.1186/1472-6963-14-2 PMC3909454

[6] Homer C Woodall J Freeman C , et al. Changing the culture: a qualitative study exploring research capacity in local government. BMC Public Health 2022;22:1341. 35836209 10.1186/s12889-022-13758-w PMC9281003

[7] Cheetham M Redgate S Van der Graaf P , et al. ‘What I really want is academics who want to partner and who care about the outcome’: findings from a mixed-methods study of evidence use in local government in England. Evid Policy 2023;19:74–94.

[8] Kneale D Rojas-García A Thomas J . Obstacles and opportunities to using research evidence in local public health decision-making in England. Health Res Policy Syst 2019;17:61. 31248422 10.1186/s12961-019-0446-x PMC6598344

[9] Van den Broucke S . Theory-informed health promotion: seeing the bigger picture by looking at the details. Health Promot Int 2012;27(2):143–7. 10.1093/heapro/das018 22581480

[10] Fleming M-L Baldwin L . Health Promotion in the 21st century: new approaches to achieving health for all. London: Routledge; 2020.

[11] Wiggers J Sanson-Fisher R . Evidence-based health. In: D Scott R Weston (eds). Evaluating health promotion. Cheltenham: Nelson Thornes; 1998. pp. 126–46.

[12] Armstrong R Waters E Dobbins M , et al. Knowledge translation strategies to improve the use of evidence in public health decision making in local government: intervention design and implementation plan. Implement Sci 2013;8:121. 24107358 10.1186/1748-5908-8-121 PMC3853093

[13] Jenkins LM Bramwell D Coleman A , et al. Integration, influence and change in public health: findings from a survey of Directors of Public Health in England. J Public Health 2016;38(3):e201–8. 10.1093/pubmed/fdv139 26487701

[14] Cheetham M Wiseman A Khazaeli B , et al. Embedded research: a promising way to create evidence-informed impact in public health? J Public Health 2018;40:i64–70. 10.1093/pubmed/fdx125 29538721

[15] Potts AJ Nobles J Shearn K , et al. Embedded researchers as part of a whole systems approach to physical activity: reflections and recommendations. Systems 2022;10:69.

[16] Cooke J . Building research capacity for impact in applied health services research partnerships comment on ‘Experience of health leadership in partnering with university-based researchers in Canada – a call to “re-imagine” research’. Int J Health Policy Manag 2021;10:93–7. 10.15171/ijhpm.2020.11 PMC7947663 32610775

[17] Cooke J Gardois P Booth A . Uncovering the mechanisms of research capacity development in health and social care: a realist synthesis. Health Res Policy Syst 2018;16:93. 30241484 10.1186/s12961-018-0363-4 PMC6150992

[18] Ritchie J . The applications of qualitative methods to social research. In: Ritchie J Lewis J (eds). Qualitative research practice. London: Sage; 2003. pp. 24–46.

[19] Cooke J . A framework to evaluate research capacity building in health care. BMC Fam Pract 2005;6:44. 16253133 10.1186/1471-2296-6-44 PMC1289281

[20] Le Gouais A Foley L Ogilvie D , et al. Decision-making for active living infrastructure in new communities: a qualitative study in England. J Public Health 2019;42:e249–58. 10.1093/pubmed/fdz105 PMC7435215 31565741

[21] Ige-Elegbede J Pilkington P Bird EL , et al. Exploring the views of planners and public health practitioners on integrating health evidence into spatial planning in England: a mixed-methods study. J Public Health 2021;43:664–72. 10.1093/pubmed/fdaa055 PMC8458017 32424415

[22] Carey G Malbon E Crammond B , et al. Can the sociology of social problems help us to understand and manage ‘lifestyle drift’? Health Promot Int 2017;32:755–61. 10.1093/heapro/dav116 26747659

[23] Brackley J Tuck P Exworthy M . Public health interventions in English local authorities: constructing the facts, (re)imagining the future. Account Audit Accoun 2021;34:1664–91.

[24] Green J Cross R Woodall J et al. Health promotion: planning and strategies. 3rd edn. London: Sage; 2019.

[25] Taylor L Haynes P Darking M . English local government finance in transition: towards the ‘marketization of income’. Public Manag Rev 2021;23:1081–106.

